# Dynamic effects of sleep deprivation on emotional behavior, circadian rhythm genes, and inflammatory infiltration in the medial prefrontal cortex

**DOI:** 10.3389/fnbeh.2025.1742898

**Published:** 2026-01-13

**Authors:** Dandan Cao, Xue Geng, Shaoqiong Yi, Haifeng Zhang, Yong Fu

**Affiliations:** 1 The Affiliated Hospital of Jiangxi University of Chinese Medicine, Nanchang, China; 2 Jiangxi University of Chinese Medicine, Nanchang, Jiangxi, China; 3 Guangzhou University of Chinese Medicine, Guangzhou, China; 4 Guangzhou City Construction College, Guangzhou, China; 5 The Affiliated Brain Hospital of Guangzhou Medical University, Guangzhou, China

**Keywords:** circadian clock genes, emotional behaviors, neuroinflammation, Sirt6/Hmgb1 pathway, sleep deprivation

## Abstract

**Background:**

Sleep, a core circadian rhythm, maintains physiological homeostasis. Its dysfunction links to neuropsychiatric disorders. Clinically, poor sleep impairs positive emotions and enhances negative emotion susceptibility, but the mechanism remains unclear, potentially involving circadian clock genes and neuroinflammatory pathways.

**Methods:**

Divide the male C57BL/6J mice into the following five groups: Non-sleep deprivation (SD) control (CON), sleep recovery 14-day after SD 7-day (SD7R14), sleep recovery 21-day after SD 7-day (SD7R21), sleep recovery 14-day after SD 14-day (SD14R14), and sleep recovery 21-day after SD 14-day (SD14R21). Behavioral tests evaluated anxiety-like behaviors, fear and andanhedonia. Histological staining observed neuronal morphology in the medial prefrontal cortex (mPFC), and RT-qPCR was employed to measure mRNA levels of circadian clock genes, Silent information regulator 6 (*Sirt6*), High mobility group box-1 (*Hmgb1*), and inflammatory factors.

**Results:**

SD induces time-dependent anxiety-like behaviors (reduced exploratory activity in elevated mazes), anhedonia (decreased sucrose preference), and fear behaviors (prolonged immobility in forced swim and tail suspension tests). Histological analysis reveals reversible neuronal damage in the mPFC, with complete recovery observed after 21 days of sleep restitution. Molecular analyses show dysregulation of the muscle aryl-hydrocarbon receptor nuclear translocator-like 1 (*Bmal1*) and circadian locomotor output cycles kaput (*Clock*) circadian pathway and activation of the Sirt6/Hmgb1 inflammatory axis, leading to proinflammatory cytokine release (*TNFα, IL1β, COX-2, IL6*), with partial recovery after sleep restoration.

**Conclusion:**

SD for 7-day or 14-day may impair emotional behaviors by disrupting the RNA expression of clock genes and the Sirt6/Hmgb1 inflammatory axis, while sleep recovery for 14-day or 21-day can partially reverse this impairment.

## Graphical abstract

**Figure:**
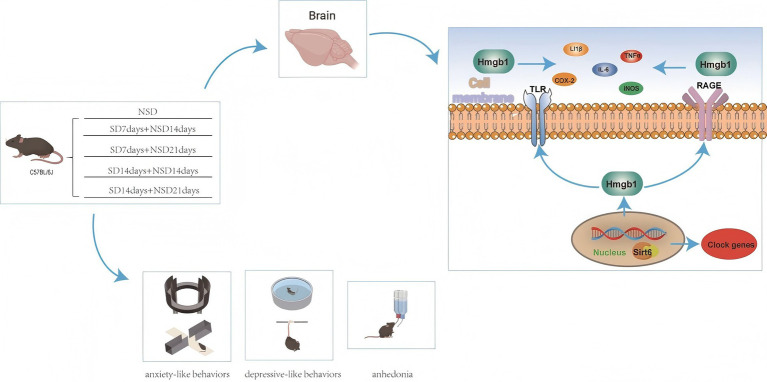

## 1 Introduction

Sleep represents a core biological rhythm modulated by the central nervous system (CNS), which sustains the body’s physiological homeostasis through circadian periodicity. Dysfunction of sleep is closely associated with neuropsychiatric disorders (Gruber and Cassoff, 2014). Clinical observations have demonstrated that poor sleep quality or insufficient sleep duration impairs positive affect and elevates an individual’s susceptibility to negative emotions (Stenson et al., 2021), yet the underlying molecular mechanisms remain elusive. Recently, a growing number of research has focused on the crosstalk between circadian clock genes and neuroinflammatory pathways, aiming to unravel the intrinsic mechanisms through which sleep modulates emotional states.

Clock genes serve as the molecular foundation governing the endogenous 24-h timing system in mammals. Beyond orchestrating the body’s circadian rhythms, these genes also play pivotal roles in maintaining sleep homeostasis, regulating energy metabolism, and modulating immune system function (Fagiani et al., 2022). The molecular core of circadian rhythms relies on the transcription-translation feedback loop (TTFL): the core clock genes brain and muscle aryl-hydrocarbon receptor nuclear translocator-like 1 (Bmal1) and circadian locomotor output cycles kaput (Clock) drive the expression of downstream target genes via binding to E-box regulatory elements. In contrast, the Period (Per1–3) and cryptochrome (Cry1–2) gene families exert negative feedback to suppress the transcriptional activity of Bmal1/Clock heterodimers. Additionally, a secondary regulatory loop consisting of retinoic acid receptor-related orphan receptors (RORα/*β*/*γ*) and nuclear receptor subfamily 1 group D members (REV-ERBα/β) fine-tunes the rhythmic expression of Bmal1, ensuring the circadian rhythm is stabilized at a 24-h cycle (Patke et al., 2019). As a typical circadian rhythm disruptor, sleep deprivation (SD) can alter the expression levels of 80% of brain circadian rhythm-related genes (Maret et al., 2007), while extensively impairing the functions of multiple key brain regions, including the hypothalamus, amygdala, corpus callosum, caudate nucleus, hippocampus, and medial prefrontal cortex (mPFC), thereby disrupting the normal physiological functions of the central nervous system (Ni et al., 2024). The mPFC acts as a core hub for emotional regulation: it governs emotional homeostasis, and per existing studies, clock genes whose expression rhythms directly modulate anxiety, fear, and other negative emotion-related behavioral phenotypes (Sarrazin et al., 2024).

Inflammation plays a critical role in sleep-emotion interactions. Silent information regulator 6 (Sirt6), an NAD^+^-dependent deacetylase, regulates chromatin remodeling and participates in aging, metabolism, and inflammation (Kugel and Mostoslavsky, 2014; Guo et al., 2022), with its dysfunction closely linked to neuroinflammation of sleep disorder patients. High-mobility group box 1 (Hmgb1), a key late-stage inflammatory molecule, induces microglial polarization and cytokine release by binding to TLR/RAGE receptors (Chen et al., 2022). Sirt6 mediates Hmgb1 translocation via deacetylation, thereby regulating inflammatory responses (Kong et al., 2020).

This study set two core objectives. First, it clarifies the dynamic effects of 7-day and 14-day SD on emotional behaviors, including anxiety-like behaviors, fear and anhedonia. It also explores whether the impairments caused by SD persist after 14-day and 21-day sleep recovery. Second, it elucidates the roles of clock genes and the Sirt6/Hmgb1 pathway in SD-induced neuroinflammation within the mPFC. It further analyzes the impact of the sleep recovery period on this process.

## 2 Materials and methods

### 2.1 Chemicals and reagents

H&E Staining Solution (C0105S) and Nissl Staining Solution (C0117) were purchased from Beyotime Institute of Biotechnology (Shanghai, China). Neutral resin (BL704A) was purchased from Biosharp Life Sciences (Hefei, China). RNAex Pro Reagent (AG21102), Evo M-MLV RT Premix (AG11706), and the SYBR® Green Premix Pro Taq HS qPCR Kit (AG11701) were obtained from Accurate Biology (Changsha, China).

### 2.2 Experimental animals and grouping

Male C57BL/6J mice (7–8 weeks old, 20–22 g) were purchased from the Animal Experiment Center of Guangzhou University of Chinese Medicine. The mice were housed in a controlled environment (22 ± 2 °C, 55 ± 5% relative humidity) under a 12-h light/dark cycle (lights on at 8:00 a.m. and off at 8:00 p.m.), with free access to food and water. All animal experiments were conducted in compliance with the ARRIVE guidelines and the National Institutes of Health Guide for the Care and Use of Laboratory Animals (NIH Publication No. 80–23, revised 1978).

After 1 week of acclimatization, the mice were randomly divided into five groups for SD (Bowers et al., 2021), with 6–7 mice per group:

(1) Non-SD control group(CON): Normal housing with normal sleep;
(2) SD7R14 group: SD for 7-day followed by 14-day of sleep recovery;
(3) SD7R21 group: SD for 7-day followed by 21-day of sleep recovery;
(4) SD14R14 group: SD for 14-day followed by 14-day of sleep recovery;
(5) SD14R21 group: SD for 14-day followed by 21-day of sleep recovery.

**Figure 1:**
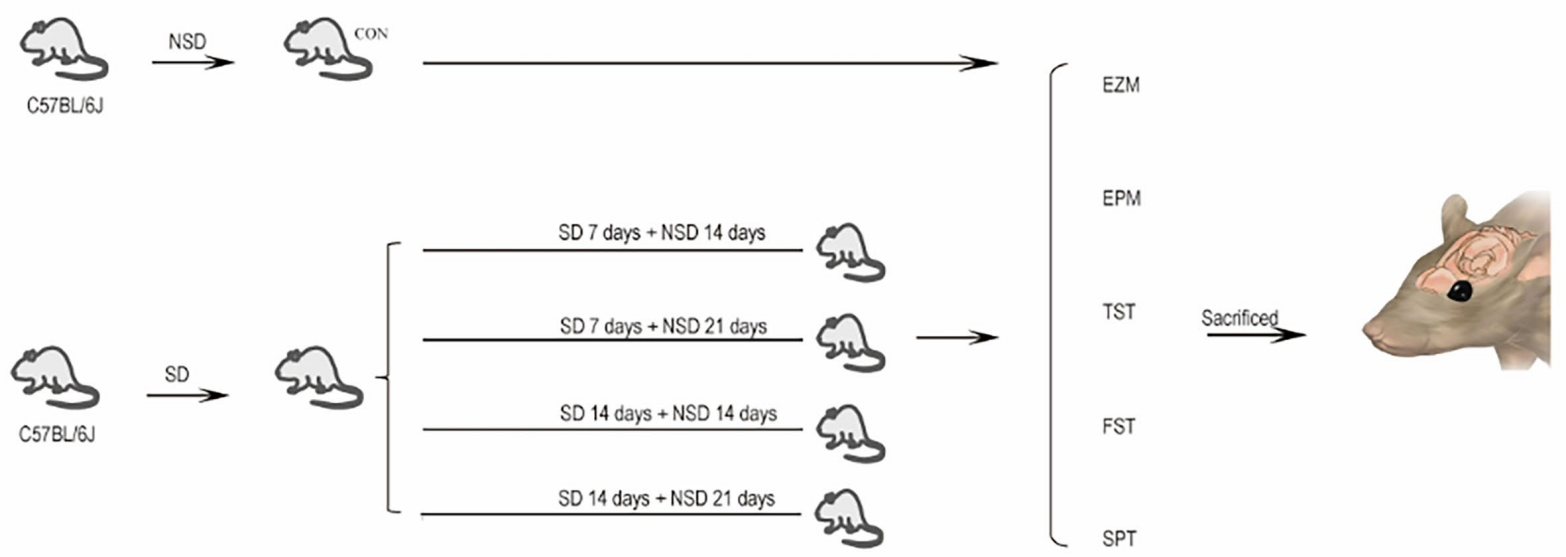
Experimental design.

On the first day of the experiment, the mice were allowed unrestricted activity from 8:00 a.m. to 12:00 p.m. SD was then implemented from 12:00 p.m. (Day 1) to 8:00 a.m. (Day 2) using a rotating drum apparatus (XR-XS108-1, Xinruan Information Technology, Shanghai, China), with the drum rotating at 2 rpm to prevent the mice from remaining immobile. The experimental design is illustrated in Figure 1.

### 2.3 Behavioral assessments

All behavioral tests were conducted between 09:30 and 11:30 a.m. during the light phase to minimize confounding effects of circadian rhythm fluctuations.

#### 2.3.1 Elevated zero maze (EZM)

The EZM apparatus comprised a circular platform (50 cm in diameter) with an open perimeter (5 cm wide) and enclosed segments with 20-cm high walls. Each mouse was placed at the center of the maze, and its locomotor activity was monitored for 5 min using a video-tracking system (XR-XZR209, Xinruan Information Technology, Shanghai, China). The time spent in the open perimeter and the entries into the open perimeter were quantified as core indices of anxiety-like behavior.

#### 2.3.2 Elevated plus maze (EPM)

The EPM consisted of two opposing open arms (35 × 5 cm, devoid of enclosures) and two opposing closed arms (35 × 5 cm, with 15-cm high walls), elevated 55 cm above the ground. Mice were subjected to a 5-min trial, and the percentages of time spent in and entries into the open arms were automatically recorded using a video-tracking software (XR-XG201, Xinruan Information Technology, Shanghai, China).

#### 2.3.3 Sucrose preference test (SPT)

Prior to testing, mice were habituated to 1% sucrose solution for 48 h, followed by a 24-h water deprivation period. In the test phase, each mouse was given simultaneous access to 1% sucrose solution and tap water for 12 h. Sucrose preference was calculated using the following formula: Preference for Sucrose (%) = (Sucrose solution consumption)/[(Sucrose solution consumption) + (Tap water consumption)] × 100%.

#### 2.3.4 Tail suspension test (TST)

Mice were suspended by the distal portion of their tails at a height of 50 cm above the floor, within a transparent chamber (55 × 15 × 11.5 cm). A side-mounted video recorder (XR-XQ203, Xinruan Information Technology, Shanghai, China) was used to record the 5-min trial, and the cumulative immobility time was quantified as a measure of behavioral despair in response to acute stress.

#### 2.3.5 Forced swimming test (FST)

Each mouse was individually placed in a cylindrical tank (10 cm in diameter) filled with water (30 ± 1 °C) to a depth of 17 cm. The trial lasted for 5 min, and a side-mounted video tracking system (XR-XQ202, Xinruan Information Technology, Shanghai, China) was employed to analyze the cumulative immobility time during the final 5-min of the test session.

### 2.4 Brain tissue collection and processing

Following the completion of all behavioral tests, mice were fasted for 12 h with ad libitum access to water. Subsequently, mice were anesthetized via intraperitoneal injection of 1% sodium pentobarbital solution, followed by euthanasia for brain tissue collection. For histological analysis, mice were transcardially perfused with PBS followed by 4% paraformaldehyde. Brains were then post-fixed and embedded in paraffin, and 4-μm coronal sections of the mPFC were prepared. For molecular biological analysis, mPFC tissues were rapidly dissected, snap-frozen in liquid nitrogen, and stored at −80 °C until subsequent use.

### 2.5 Histological and molecular analyses

#### 2.5.1 H&E and Nissl staining

Paraffin sections were deparaffinized, rehydrated, and stained with H&E or Nissl staining. Images were captured using a pathological scanner (KF-PRO-005, KFBIO, Guangzhou, China). Neuronal morphology, nuclear pyknosis, and Nissl body density were evaluated based on the acquired images.

#### 2.5.2 RT-qPCR analysis

Total RNA was extracted from mouse mPFC tissues using Trizol reagent and RNAex Pro Reagent. The concentration and purity of RNA samples were determined using a NanoPhotometer (NP80, Implen, Germany) by measuring the absorbance ratio at 260/280 nm. Reverse transcription was performed in a 10 μL reaction volume containing 500 ng of total RNA using the Evo M-MLV RT Premix Kit. qPCR was conducted on the synthesized cDNA using the SYBR® Green Premix Pro Taq HS qPCR Kit with a CFX Connect Real-Time PCR Detection System (Bio-RAD, CA, USA). The qPCR program consisted of 40 cycles: initial denaturation at 95 °C for 30 s, followed by denaturation at 95 °C for 5 s and annealing/extension at 60 °C for 30 s. The resulting data were analyzed using the formula E = 2^-ΔΔCt^. Primers were designed by the first author under the supervision of Sangon Biotech (Shanghai, China). Detailed primer sequences are provided in Table 1.

**Table 1:** Primer sequences for RT–qPCR.

Gene	Forward primer sequence	Reverse primer sequence
*Sirt6*	CCCAAGTGTAAGACGCAGTA	GTCCAGAATGGTGTCTCTCAG
*Hmgb1*	AGGCTGACAAGGCTCGTTATGAAAG	GGGCGGTACTCAGAACAGAACAAG
*TNF-α*	GAGTCCGGGCAGGTCTACTTT	CAGGTCACTGTCCCAGCATCT
*IL-6*	CCAGAAACCGCTATGAAGTTC	CCACCAGCATCAGTCCCAAGA
*IL-1β*	CCTCGTGCTGTCGGACCCATA	CCTCGTGCTGTCGGACCCATA
*COX-2*	TGGAGATCATGGGGAGTCTG	AAGAAAACCTGGTCCGGTGAA
*Bmal1*	GGACTTCGCCTCTACCTGTTCAAAG	TCGTTGTCTGGCTCATTGTCTTCG
*Clock*	TGGTGACTGCCTATCCTACCTTCG	TGCTGCTGCTGCTGCTGTTG
*Per1*	CCTGGGCTCTGGGTCTGGTTC	TTGCTTGTATGGCTGCTCTGACTG
*Per2*	GCTGCGGATGCTCGTGGAATC	GGTTGTGCTCTGCCTCTGTCATC
*Per3*	AAAGATCCTGACCTCGCCCTACG	GTGCTTCTGCCTCTCGCTTCC
*Cry1*	GCCAGCAGACACCATCACATCAG	CCAGGGAAGGAACGCCATATTTCTC
*Cry2*	TGGACAAGCACTTGGAACGGAAG	GTAGAAGAGGCGGCAGGAGAGG
*β-actin*	CCTCTATGCCAACACAGTGC	GTACTCCTGCTTGCTGATCC

### 2.6 Statistical analysis

Behavioral data were analyzed using VisuTrack Animal Behavior Analysis Software (XR-VT, Xinruan Information Technology, Shanghai, China). All data are presented as the mean ± standard error of the mean (SEM) and processed using GraphPad Prism 9 software (GraphPad Software, Inc., La Jolla, CA, USA). For numerical data, statistical comparisons between groups were performed using one-way analysis of variance (ANOVA) followed by post-hoc tests or non-parametric tests for unpaired observations, as appropriate. A *p*-value ≤ 0.05 was considered statistically significant for all analyses.

## 3 Results

### 3.1 SD induces time-dependent anxiety-like behaviors

**Figure 2:**
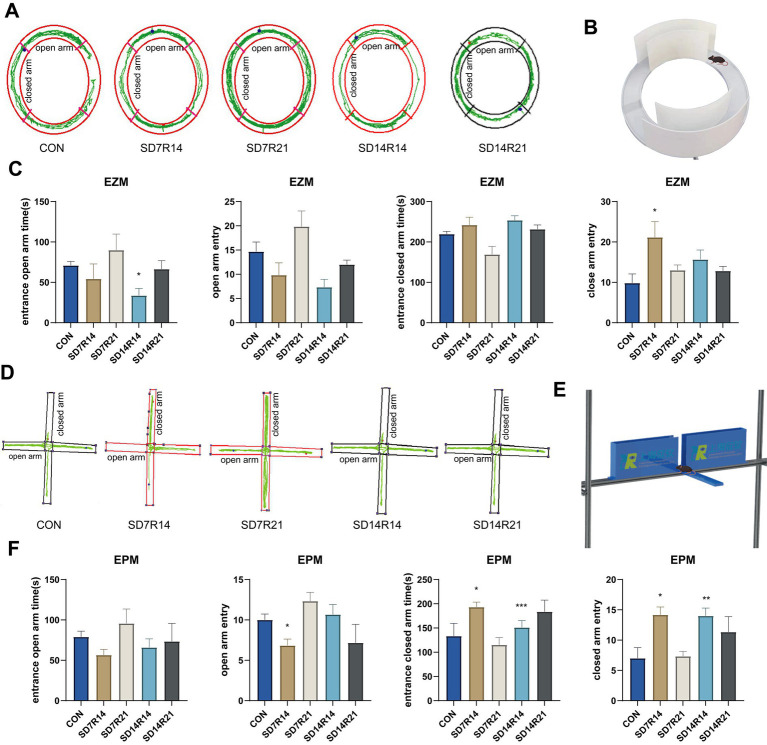
SD-induced anxiety-like behaviors. **(A)** Representative movement trajectories of mice in the EZM following SD. **(B)** EZM experiment diagram. **(C)** Quantification of time spent and entry frequencies in open or closed arms of the EZM. **(D)** Movement trajectories of mice in the EPM after SD. **(E)** EPM experiment diagram. **(F)** Time and entry counts in open or closed arms of the EPM. Data are presented as mean ± SEM (*n* = 6). *p ≤ 0.05, compared to the CON group. EZM, Elevated zero maze; EPM, elevated plus maze; SD, sleep deprivation; R, sleep recovery.

Anxiety-like behaviors were assessed by quantifying the duration of residence and frequency of entries in the open versus closed arms of the EZM and EPM following SD in mice(Kim et al., 2025). In the EZM, relative to the CON group, mice in the SD7R14, SD14R14, and SD14R21 cohorts exhibited reductions in both the time spent in open arms (η^2^ = 0.268, *p* > 0.05, *p* ≤ 0.05, and *p* > 0.05, Figures 2A–C) and the frequency of open-arm entries (η^2^ = 0.432, all *p* > 0.05, Figures 2A–C). Conversely, the SD7R21 group displayed increases in both open-arm residence time (η^2^ = 0.268, *p* > 0.05, Figures 2A–C) and entry frequency (η^2^ = 0.432, *p* > 0.05, Figures 2A–C). For closed-arm metrics, the SD7R14, SD14R14, and SD14R21 groups showed elevated residence time (η^2^ = 0.45, all *p* > 0.05, Figures 2A–C) and entry frequency (η^2^ = 0.339, *p* ≤ 0.05, *p* > 0.05, and *p* > 0.05, Figures 2A–C). The SD7R21 group, by contrast, had shortened closed-arm residence time (η^2^ = 0.45, *p* > 0.05, Figures 2A–C) but increased closed-arm entry frequency (η^2^ = 0.339, *p* > 0.05, Figures 2A–C). Notably, the SD14R14 group demonstrated the most pronounced decreases in open-arm time and entry counts, while the SD7R14 group exhibited the highest closed-arm residence duration and entry frequency.

In the EPM assay, compared with the CON group, the SD7R14, SD14R14, and SD14R21 groups showed reduced open-arm residence time (η^2^ = 0.144, all *p* > 0.05, Figures 2D–F). The SD7R14 and SD14R21 groups also had fewer open-arm entries (η^2^ = 0.326, *p* ≤ 0.05 and *p* > 0.05, Figures 2D–F), whereas the SD14R14 group displayed a trend toward increased open-arm entry frequency (η^2^ = 0.326, *p* > 0.05, Figures 2D–F). In contrast, the SD7R21 group exhibited increases in both open-arm residence time (η^2^ = 0.144, *p* > 0.05, Figures 2D–F) and entry frequency (η^2^ = 0.326, *p* > 0.05, Figures 2D–F). For closed-arm parameters, the SD7R14, SD14R14, and SD14R21 groups had prolonged residence time (η^2^ = 0.326, *p* ≤ 0.05, *p* ≤ 0.001, and *p* > 0.05, Figures 2D–F) and higher entry frequency (η^2^ = 0.417, *p* ≤ 0.05, *p* ≤ 0.01, and *p* > 0.05, Figures 2D–F). The SD7R21 group showed diminished closed-arm residence time (η^2^ = 0.326, *p* > 0.05, Figures 2D–F) and a marginal elevation in closed-arm entry frequency (η^2^ = 0.417, *p* > 0.05, Figures 2D–F). Specifically, the SD7R14 and SD14R14 groups exhibited the most substantial reductions in open-arm time and entry counts, which coincided with the greatest increases in closed-arm residence duration and entry frequency. In contrast, anxiety-like behaviors were markedly attenuated following 21 days of sleep recovery after SD.

In aggregate, these findings indicate that SD impairs exploratory behavior in mice, implying that it may induce anxiety-like phenotypes in susceptible individuals. However, SD-elicited anxiety-like behaviors were gradually mitigated with the extension of normal sleep recovery periods. That said, statistical analyses across multiple groups revealed no significant differences, a limitation that may be attributable to insufficient sample sizes per group and which, to some extent, compromised the reliability of these findings. To address this caveat, we further performed a battery of behavioral assays to evaluate fear responses and anhedonia.

### 3.2 SD induces time-dependent fear and anhedonia

Following the assessment of anxiety-like behaviors, we next evaluated fear- and anhedonia -related behaviors in mice.

The FST and TST were employed to assess fear (Martins et al., 2025; Liu et al., 2021), as immobility durations in these paradigms are considered reliable indicators of anxiety-like behaviors (Yankelevitch-Yahav et al., 2015). To elucidate the impact of SD on affective behavioral alterations in mice, the FST and TST were implemented in the present study.

**Figure 3:**
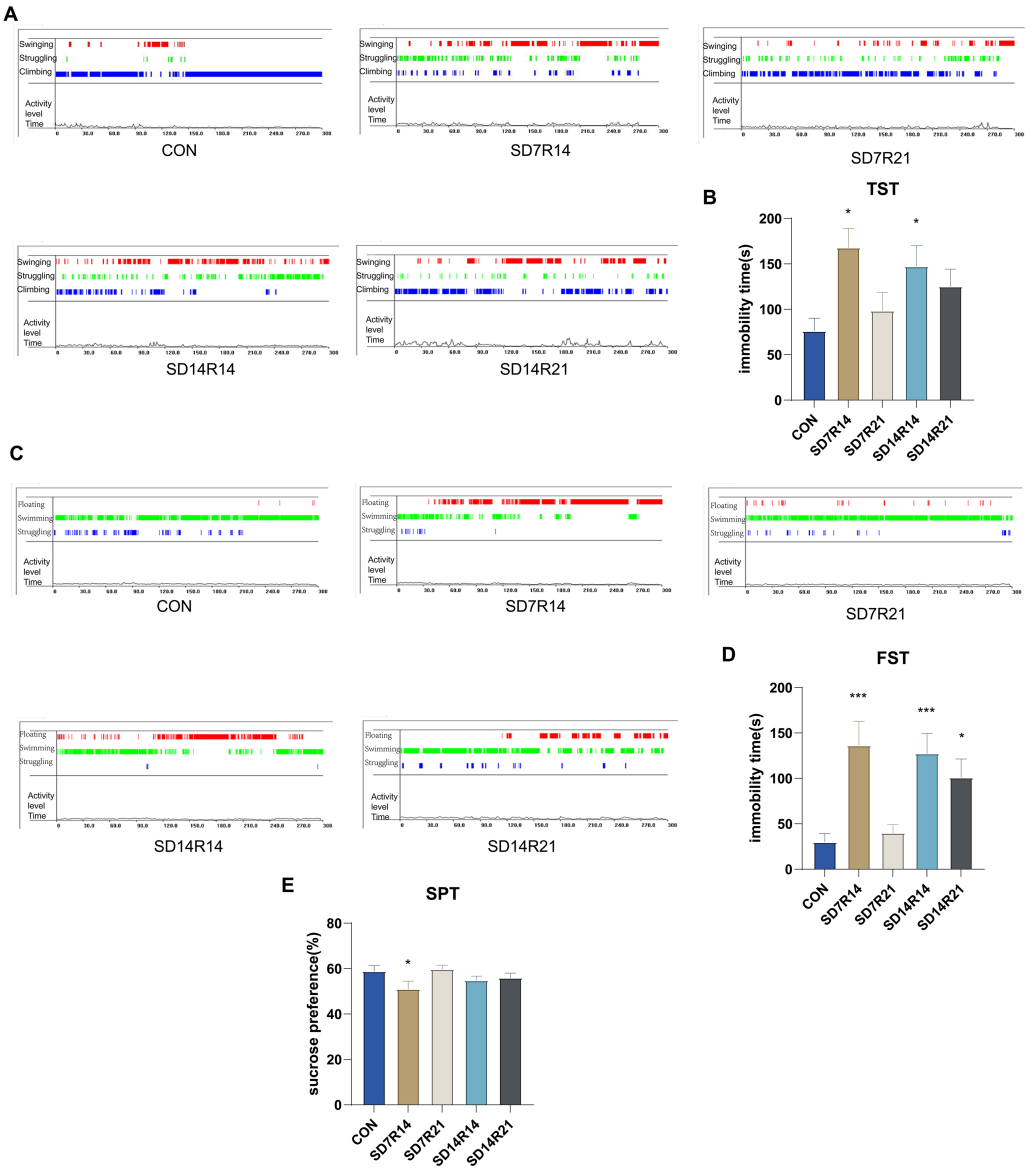
SD promotes fear and anhedonia. **(A)** Behavioral states in the TST: red = swinging, green = struggling, blue = climbing. **(B)** Immobility duration in TST. **(C)** Behavioral states in the FST: red = floating, green = swimming, blue = struggling. **(D)** Immobility duration in FST. **(E)** Sucrose preference (%) in mice. Data are presented as mean ± SEM (*n* = 6). *p ≤ 0.05, **p ≤ 0.01, and ***p ≤ 0.001, compared to the CON group. TST, Tail Suspension Test; FST, Forced Swimming Test; SPT, Sucrose Preference Test; SD, sleep deprivation; R, sleep recovery.

In the TST, compared with the CON group, mice in the SD7R14, SD7R21, SD14R14, and SD14R21 cohorts exhibited prolonged immobility durations (η^2^ = 0.358, *p* ≤ 0.05, *p* > 0.05, *p* ≤ 0.05, and p > 0.05, Figures 3A, B). Similarly, in the FST, relative to the CON group, mice in the SD7R14, SD7R21, SD14R14, and SD14R21 groups displayed increased floating immobility times (η^2^ = 0.52, *p* ≤ 0.001, *p* > 0.05, *p* ≤ 0.001, and *p* ≤ 0.05, Figures 3C, D). Notably, the SD7R14 and SD14R14 groups showed the most pronounced elevations in immobility duration, with the SD7R14 cohort demonstrating the most striking phenotype, the SD7R21 group saw very little increase.

Anhedonia, defined as a diminished capacity to experience reward or pleasure, is a core endophenotype of depressive-like states in stressed animals and is typically characterized by attenuated reward-seeking behavior (Primo et al., 2023). Herein, the sucrose preference ratio was utilized as a validated metric to assess hedonic responsiveness in mice. In the SPT, relative to the CON group, mice in the SD7R14, SD14R14, and SD14R21 groups exhibited reduced sucrose preference ratios (η^2^ = 0.25, *p* ≤ 0.05, *p* > 0.05, and *p* > 0.05, Figure 3E), whereas only the SD7R21 cohort showed an increase in this index (η^2^ = 0.25, *p* > 0.05, Figure 3E).

Taken together, these findings demonstrate that SD can elicit fear-like behaviors and anhedonia in susceptible mice, and these pathological phenotypes are amenable to reversal with adequate sleep recovery duration.

### 3.3 SD causes brian damage in the mPFC

**Figure 4:**
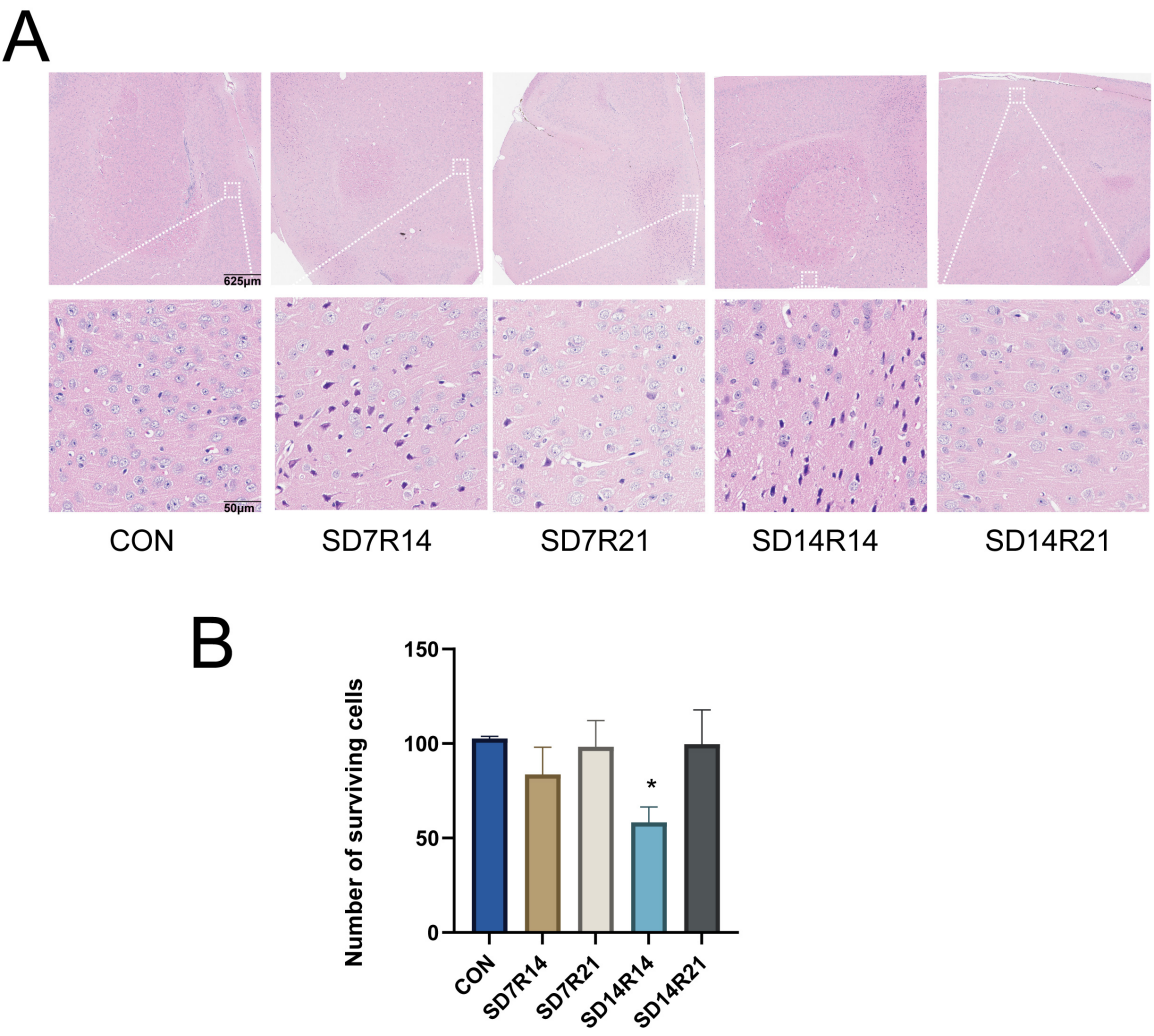
SD-induced brain tissue damage. **(A)** Representative H&E staining of the mPFC. **(B)** Cell count chart. Data are presented as mean ± SEM (*n* = 3). *p ≤ 0.05, compared to the CON group. SD, sleep deprivation; R, sleep recovery.

SD induced structural damage to brain tissue and neuronal degeneration in the mPFC, a brain region critical for affective and cognitive regulation. As depicted in Figure 4A, compared with the (CON) group, mice in the SD7R14 and SD14R14 cohorts exhibited prominent expansion of intercellular spaces in the mPFC, accompanied by nuclear atrophy and disorganized cellular arrangement. In contrast, no such overt pathological alterations were observed in the SD7R21 and SD14R21 groups. Quantitative analysis of viable cell counts within equivalent anatomical areas further revealed that the SD7R14 and SD14R14 groups had fewer surviving mPFC neurons relative to the CON group (η^2^ = 0.459, *p* > 0.05 and *p* ≤ 0.05, Figure 4B), whereas cell viability in the SD7R21 and SD14R21 groups did not differ significantly from the CON group (η^2^ = 0.459, all *p* > 0.05, Figure 4B).

**Figure 5:**
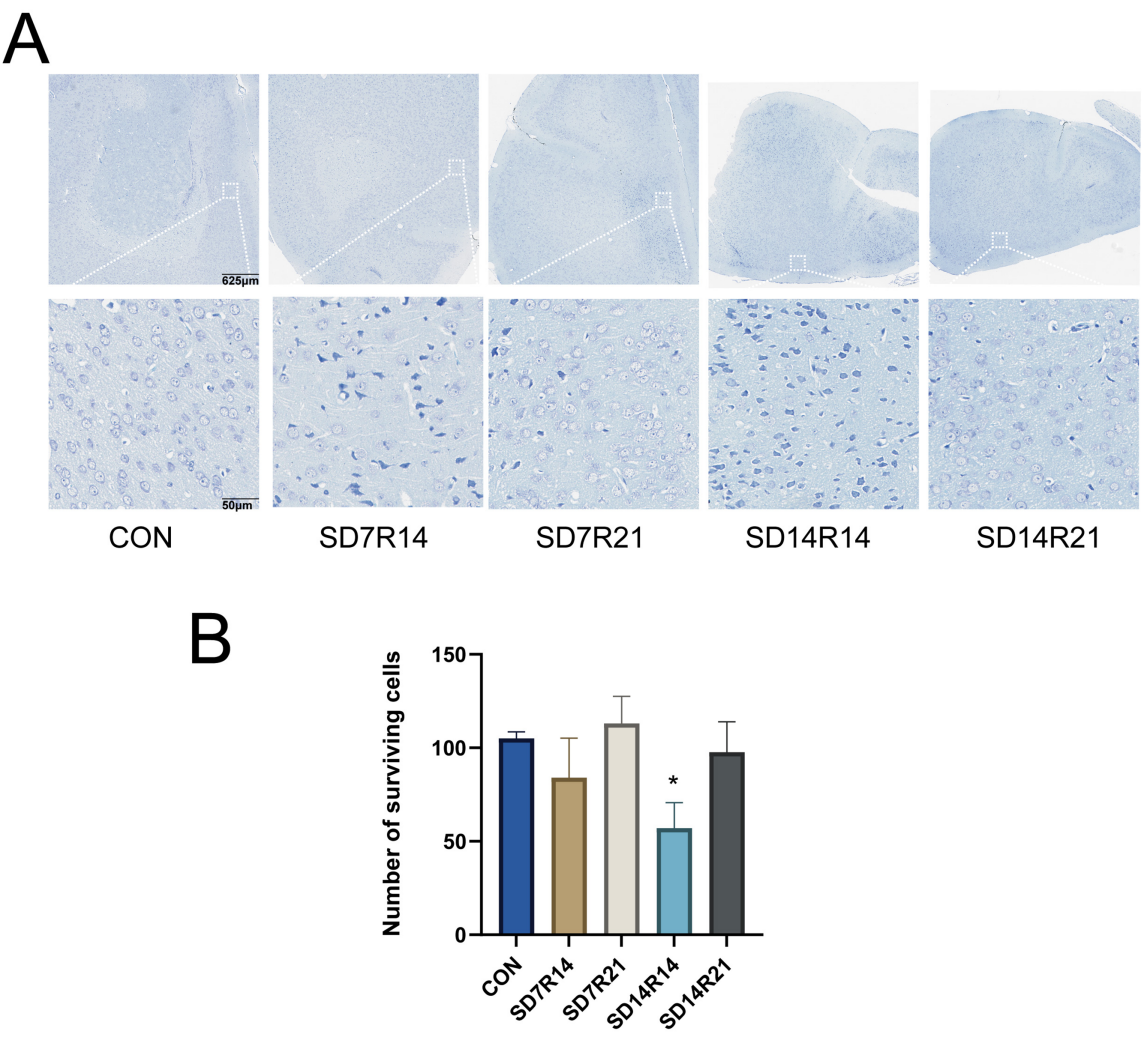
Enhanced neuronal injury in mPFC. **(A)** Representative Nissl staining of mPFC neurons of the mPFC. **(B)** Cell count chart. Data are presented as mean ± SEM (*n* = 3). *p ≤ 0.05, compared to the CON group. SD, Sleep deprivation; R, Sleep recovery.

To further characterize neuronal morphological perturbations in the mPFC, Nissl staining was performed. As illustrated in Figure 5A, relative to the CON group, the SD7R14 and SD14R14 cohorts displayed partial loss of Nissl bodies, neuronal soma shrinkage or disintegration, nuclear pyknosis with hyperchromasia, and blunted neuronal contours in the mPFC. Notably, these pathological features were absent in the SD7R21 and SD14R21 groups. Consistent with the general histological findings, quantitative cell counting in matched regions demonstrated reduced viable neuron numbers in the SD7R14 and SD14R14 groups compared with the CON group (η^2^ = 0.463, *p* > 0.05 and *p* ≤ 0.05, Figure 5B), while the SD7R21 and SD14R21 groups showed no substantial deviations from the CON group (η^2^ = 0.463, all *p* > 0.05, Figure 5B).

Altogether, these histopathological and morphometric findings indicate that SD elicits brain tissue injury and neuronal degeneration in the mPFC, and these deleterious effects are reversible following a sufficient period of sleep recovery. Importantly, the severity of these structural alterations was correlated with the duration of SD exposure and the length of subsequent sleep restoration, highlighting a time-dependent neuroprotective role of sleep recovery against SD-induced neural damage.

### 3.4 SD interferences clock genes expression

**Figure 6:**
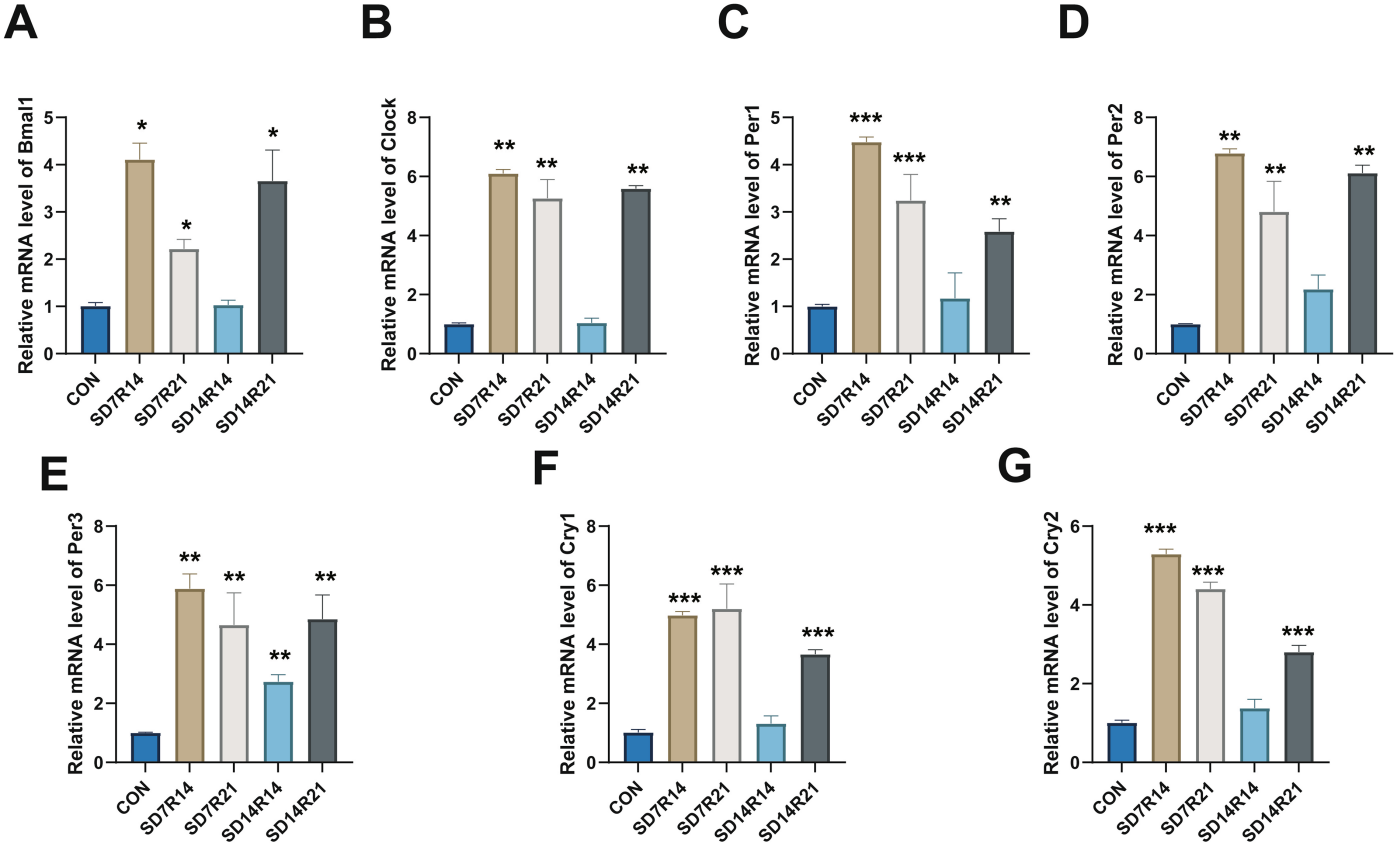
Dysregulated clock genes expression in SD mice. **(A–G)** RT-qPCR analysis of mPFC clock genes (*Bmal1, Clock, Per1, Per2, Per3, Cry1, Cry2*) relative expression. Data are mean ± SEM (*n* = 6). *p ≤ 0.05, **p ≤ 0.01, and ***p ≤ 0.001, compared to the CON group. SD, Sleep deprivation; R, Sleep recovery.

The sleep–wake cycle represents a key output modality of the circadian clock system, and SD-induced disruption of this cycle is known to elicit dysregulation of clock gene transcription—a phenomenon corroborated by the findings of the present study. Compared with the naive control group, the mRNA expression levels of *Bmal1* were significantly elevated in the SD7R14, SD7R21, and SD14R21 groups (η^2^ = 0.735, all *p* ≤ 0.05, Figure 6A), whereas no markedly increased was observed in the SD14R14 group (η^2^ = 0.735, *p* > 0.05; Figure 6A). A similar expression pattern was noted for *Clock*: its transcript levels were markedly upregulated in the SD7R14, SD7R21, and SD14R21 groups (η^2^ = 0.918, all *p* ≤ 0.01, Figure 6B), with the SD14R14 group showing no discernible deviation from controls (η^2^ = 0.918, *p* > 0.05; Figure 6B).

For the *Per* gene family, *Per1* mRNA levels were robustly increased in the SD7R14 and SD7R21 groups (η^2^ = 0.714, both *p* ≤ 0.001, Figure 6C), moderately elevated in the SD14R21 group (*p* ≤ 0.01), and unchanged in the SD14R14 group (η^2^ = 0.714, *p* > 0.05; Figure 6C). Concordantly, *Per2* expression was significantly augmented in the SD7R14, SD7R21, and SD14R21 groups (η^2^ = 0.785, all *p* ≤ 0.01, Figure 6D) but comparable to controls in the SD14R14 cohort (η^2^ = 0.785, *p* > 0.05; Figure 6D). Notably, *Per3* exhibited uniform upregulation across all SD-exposed groups (η^2^ = 0.584, all *p* ≤ 0.01; Figure 6E), distinguishing it from other Per paralogs by its lack of a compensatory return to baseline in the SD14R14 group.

With respect to *Cry* genes, *Cry1* transcript abundance was drastically increased in the SD7R14, SD7R21, and SD14R21 groups (η^2^ = 0.791, all *p* ≤ 0.001; Figure 6F), while the SD14R14 group showed no significant alteration (η^2^ = 0.791, *p* > 0.05; Figure 6F). This trend was mirrored by Cry2, whose expression was profoundly elevated in the SD7R14, SD7R21, and SD14R21 groups (η^2^ = 0.955, all *p* ≤ 0.001, Figure 6G) but indistinguishable from controls in the SD14R14 group (η^2^ = 0.955, *p* > 0.05; Figure 6G). Across all clock genes examined, the most pronounced transcriptional upregulation was consistently observed in the SD7R14 group, whereas the SD14R14 group displayed near-baseline expression profiles overall.

Collectively, these data demonstrate that SD perturbs the canonical expression dynamics of core clock genes in mice, with such perturbations persisting even after 21 days of recovery sleep. Notably, the restoration of clock gene expression to control-like levels in the SD14R14 group suggests that prolonged SD may trigger compensatory transcriptional rewiring of the circadian oscillator. However, the loss of this compensatory transcriptional remodeling following 21 days of recovery implies that the clock system adaptive capacity is time-limited.

### 3.5 SD regulates pro-inflammatory cytokine expression in the mPFC via the Sirt6/Hmgb1 pathway

SD can activate glial cells and trigger the release of pro-inflammatory cytokines, among which the Sirt6/Hmgb1 signaling pathway plays a core regulatory role in initiating inflammatory responses within the mPFC. To clarify the regulatory mechanism of the Sirt6/Hmgb1 pathway on pro-inflammatory cytokines after SD, this study detected the mRNA expression levels of Sirt6, Hmgb1, and key pro-inflammatory factors in the mPFC.

**Figure 7:**
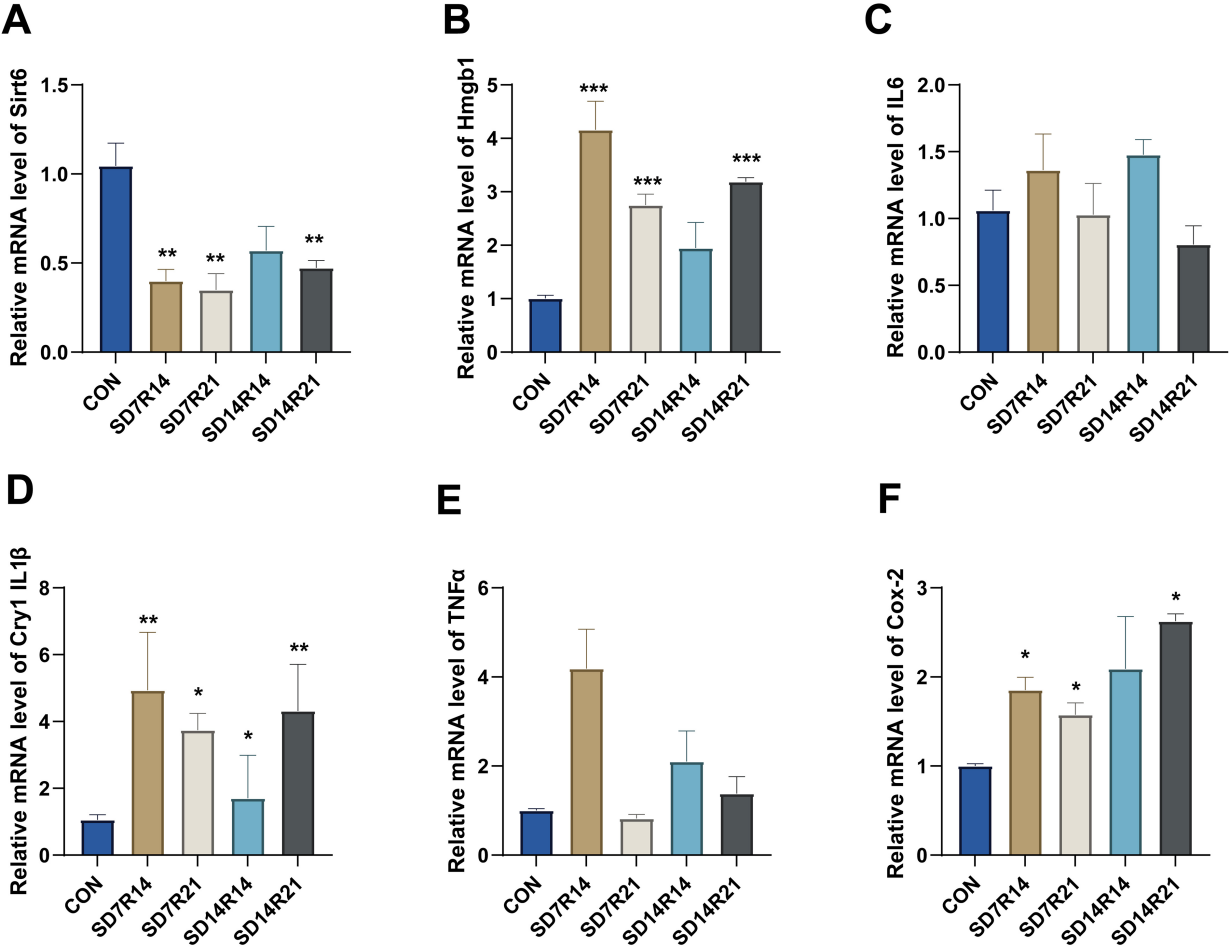
Sirt6/Hmgb1 pathway mediates neuroinflammatory response in SD mice. **(A,B)** RT-qPCR analysis of *Sirt6* and *Hmgb1* mRNA levels. **(C–F)** Relative expression of proinflammatory factors (*IL6, IL1β, TNFα, and Cox-2*) measured by RT-qPCR. Data are mean ± SEM (*n* = 6). *p ≤ 0.05, **p ≤ 0.01, and ***p ≤ 0.001, compared to the CON group. SD, sleep deprivation; R, sleep recovery.

Compared with the CON group, the groups with different recovery periods after SD treatment showed characteristic changes in the expression of pathway molecules and pro-inflammatory cytokines. The mRNA level of *Sirt6* was significantly downregulated in all the aforementioned groups (η^2^ = 0.563), among which the downregulation was statistically significant in the SD7R14, SD7R21, and SD14R21 groups (all *p* ≤ 0.05, Figure 7A), while only the SD14R14 group did not reach a significant level (*p* > 0.05, Figure 7A). In contrast, the mRNA level of *Hmgb1* was significantly upregulated in all experimental groups (η^2^ = 0.671); except for the SD14R14 group (*p* > 0.05, Figure 7B), the upregulation in the SD7R14, SD7R21, and SD14R21 groups was extremely statistically significant (all *p* ≤ 0.001, Figure 7B). These results suggest that SD can significantly regulate the expression of core molecules in the Sirt6/Hmgb1 pathway.

At the level of pro-inflammatory factors, the mRNA level of *IL1β* showed an upward trend in all experimental groups (η^2^ = 0.418), among which the upregulation was extremely significant in the SD7R14 and SD14R21 groups (all *p* ≤ 0.01, Figure 7D), and significant in the SD7R21 and SD14R14 groups (all *p* ≤ 0.05, Figure 7D). The mRNA expression change of *Cox-2* was similar to that of *IL1β*, showing a significant upward trend in all experimental groups (η^2^ = 0.427); except for the SD14R14 group (*p* > 0.05, Figure 7F), the upregulation in the other groups was statistically significant (all *p* ≤ 0.05, Figure 7F). The mRNA level of *IL6* showed time-dependent differences: it tended to increase in the SD7R14 and SD14R14 groups, but turned to decrease in the SD7R21 and SD14R21 groups. However, none of the differences between groups reached statistical significance (η^2^ = 0.244, all *p* > 0.05, Figure 7C). The mRNA expression of *TNFα* also showed recovery time-dependent characteristics: it tended to increase in the SD7R14, SD14R14, and SD14R21 groups, while it tended to decrease in the SD7R21 group, and the differences between groups were also not statistically significant (η^2^ = 0.14, all *p* > 0.05, Figure 7E).

Notably, the overall upregulation of pro-inflammatory cytokines was most pronounced in the SD7R14 and SD14R14 groups, indicating that the early recovery phase following SD may represent the peak period of neuroinflammatory response. Although not all pro-inflammatory cytokines examined exhibited statistically significant differences, the collective data nonetheless corroborate the pro-inflammatory effects of SD. Collectively, these results demonstrate that SD activates the Sirt6/Hmgb1 signaling pathway in the mPFC by downregulating *Sirt6* expression and upregulating *Hmgb1* expression, thereby triggering the release of pro-inflammatory cytokines. This finding furnishes critical experimental evidence for deciphering the molecular underpinnings of SD-associated neuroinflammation in the mPFC.

## 4 Discussion

This study confirmed that SD could induce a series of negative emotion-related behavioral alterations in mice in a time-dependent manner, specifically manifested as follows: a significant reduction in the exploration time of the open arms in the EZM and EPM, indicating exacerbated anxiety-like behaviors; prolonged immobility time in the TST and FST, reflecting enhanced fear; and decreased sucrose preference rate in the SPT, suggesting the emergence of obvious anhedonia. Further comparison of the effects of SD with different durations revealed that the degree of negative emotional behavioral impairment in mice from the 14-day SD group was significantly more severe than that in the 7-day SD group. This finding is consistent with conclusions from clinical studies that acute SD impairs the body’s emotion regulation function, while chronic SD further increases individual emotional vulnerability (Palmer et al., 2024). Notably, after 21 days of sleep recovery intervention, the aforementioned abnormal behaviors of SD-modeled mice basically returned to normal levels. This discovery provides crucial experimental evidence for clarifying the dynamic evolution time law of negative emotional behavioral phenotypes induced by SD.

Furthermore, the recovery of behavioral indicators was correlated with the findings of histological assays: neuronal damage in brain tissues persisted in mice subjected to 7-day or 14-day SD after 14-day of sleep recovery under normal conditions; in contrast, neuronal morphology was largely restored to normal following 21-day of recovery. A distinct time lag was observed between neuronal repair and behavioral improvement. This phenomenon suggests that neuronal damage may serve as a secondary factor that delays behavioral recovery, while also providing a potential therapeutic window for sleep-targeted interventions.

Circadian rhythms govern cellular functions via the TTFL, regulating signaling cascades, metabolic homeostasis, and immune-inflammatory responses (Chaix et al., 2016). The sleep–wake cycle represents a prominent manifestation of human circadian regulation. Emerging evidence indicates a bidirectional relationship between sleep duration and clock gene expression. Individuals with a late-night sleep phenotype exhibit disrupted circadian oscillations in the mPFC, a neural hub implicated in mood regulation, which may predispose them to affective disorders (Min and Min, 2018). Genetic studies have further established that clock gene dysregulation significantly elevates the risk of developing psychological illness (Bunney et al., 2015). Insufficient sleep can induce circadian clock genes dysregulation and trigger mood disorders, yet the intrinsic link between clock gene dysregulation and emotional behavioral abnormalities remains unclear. Our study has observed that SD can induce negative emotional phenotypes in mice. Accordingly, a key question arises: does profound dysregulation occur at the level of clock genes transcription underlying these phenotypic changes?

As core TTFL components, Bmal1 and Clock act as master transcriptional regulators that drive rhythmic expression of Per1-3 and Cry1-2 genes (Wisor et al., 2008; Porcu et al., 2020). Previous studies employing clock gene-mutant or clock gene-knockout animal models have demonstrated that the ablation of a single clock gene exerts a marked impact on the TTFL underlying circadian rhythms (Ye et al., 2014), Mechanistically, the positive and negative feedback transcription of clock genes occurs initially at the DNA level, followed by events independent of the DNA template (Koike et al., 2012; Le Martelot et al., 2012). To explore how sleep–wake cycle disruption impacts circadian regulation, we conducted RT-qPCR analyses on mPFC tissues from SD mice. Collectively, our data show that SD disrupts canonical expression dynamics of core clock genes in mice, with perturbations persisting even after 21 days of recovery sleep. However, loss of this adaptive remodeling after 21 days of recovery indicates the clock system’s compensatory capacity is time-limited, likely due to depleted cellular homeostatic resources or persistent SD-induced epigenetic modifications. A key unresolved question is the biological significance of post-recovery clock gene downregulation: is it pathological or a physiological compensation?

To address this, we focus on the TTFL: Clock genes transcription relies on a bidirectional positive–negative feedback loop, and aberrant overexpression of core clock genes impairs TTFL negative feedback. Thus, we hypothesize post-recovery clock gene downregulation is a compensatory mechanism central to restoring TTFL-mediated circadian homeostasis. This hypothesis gains indirect support from a recent study on Clock gene translational regulation in sleep/circadian rhythms, which validated core clock gene transcription dynamics (Sun et al., 2025). These findings highlight the clock’s complex SD responses and the need to clarify the molecular basis of time-limited compensation.

Notably, some research has underscored the intricate interplay between circadian dysregulation, immune function, and inflammation (Deurveilher et al., 2021). As a physiological stressor, SD has been shown to trigger immune-inflammatory cascades (Fan et al., 2018). Clock genes disruptions serve as key mediators of central and peripheral inflammatory responses, a link further corroborated by the established association between circadian disturbance and a spectrum of inflammatory diseases (Liu et al., 2017; Steffens et al., 2017; Hardeland, 2019). Pro-inflammatory cytokines—including *IL1β*, *IL6*, and TNF-α, which are critical mediators of neuroinflammation—also play an integral role in the regulation of sleep homeostasis (Taishi et al., 1998; Xu et al., 2023). Consistent with these prior findings, our experimental data revealed that SD elicited a marked upregulation of *IL1β, IL6, Cox-2,* and *TNFα* expression, thereby initiating a robust inflammatory response within the murine brain. After 21 days of sleep recovery, the inflammatory response was significantly reduced.

Sirt6, an NAD^+^-dependent protein deacetylase, exerts pivotal regulatory functions in circadian biology by orchestrating local chromatin remodeling to facilitate high-amplitude transcription of core clock genes (Masri et al., 2014; Sun et al., 2019). Additionally, mounting evidence suggests that Sirt6 acts as a negative regulator of the inflammatory response by suppressing the release of proinflammatory cytokines (Simon et al., 2019; Kumar et al., 2022). However, the precise molecular pathways through which Sirt6 influences negative emotional behaviors remain largely elusive.

As a canonical damage-associated molecular pattern, Hmgb1 activates proinflammatory cascades through its interaction with two distinct signaling axes: the TLR4/NF-κB pathway and the TNF-α/TNFR1/NF-κB pathway (Xu et al., 2020). The biological functions of Hmgb1 are critically determined by its subcellular localization: in the nucleus, Hmgb1 functions as a DNA-binding protein, participating in fundamental processes such as DNA replication, transcription, and damage repair (Wang et al., 2020). Under stress conditions, Hmgb1 translocates from the nucleus to the cytoplasm, where it initiates immune responses. Recent studies have identified Sirt6 as a key regulator of Hmgb1 cytoplasmic translocation via deacetylation, a process that modulates microglial polarization and activates downstream inflammatory pathways (Le et al., 2019; Kong et al., 2020). Our study preliminarily verified the above mechanism: SD significantly downregulates the expression level of *Sirt6*, increases the expression of *Hmgb1*, further activates inflammatory signaling pathways, and ultimately leads to the release of various pro-inflammatory mediators. Previous studies have shown that circadian rhythm disruption promotes inflammatory responses. Therefore, how clock genes dysregulation and the Sirt6/Hmgb1 axis interact to regulate pro-inflammatory factor release remains a key question for future research.

## 5 Conclusion

Our study demonstrated that 7 or 14 days of SD markedly impairs mice emotion-related behaviors, an effect driven by synergistic RNA-level circadian clock gene transcriptional dysregulation and Sirt6/Hmgb1-mediated inflammatory infiltration. In contrast, 14 or 21 days of sleep recovery partially reverses these deficits, concomitant with restored clock genes RNA-transcriptional perturbations and attenuated inflammation. These findings deepen mechanistic insights into sleep-emotion crosstalk by underscoring the pivotal role of circadian transcriptional regulation and neuroinflammation, while providing critical evidence for delineating the temporal trajectory of SD-induced negative emotional phenotypes.

### 5.1 Limitations and translational implications

This study was limited to RNA-level analyses of the effects of SD on clock genes and neuroinflammation, with no corresponding investigations performed at the protein level—a key methodological constraint of the present work. Compounding this limitation, the research was restricted to a single brain region, which further impairs the comprehensiveness and generalizability of the findings. Nevertheless, this study successfully identifies two promising therapeutic targets for SD-associated pathologies: (1) targeted administration of selective agonists or inhibitors during Bmal1/Clock-mediated TTFL dysfunction could enable the artificial modulation of circadian transcriptional rhythms, thereby restoring the integrity of the circadian regulatory network; (2) Hmgb1 antagonists may serve as effective interventional agents to mitigate SD-induced neuroinflammation.

## Data Availability

The original contributions presented in the study are included in the article/Supplementary material, further inquiries can be directed to the corresponding author.
